# The role of fibromodulin in inflammatory responses and diseases associated with inflammation

**DOI:** 10.3389/fimmu.2023.1191787

**Published:** 2023-07-07

**Authors:** Feng Zhao, Yang Bai, Xuerong Xiang, Xiaoxiao Pang

**Affiliations:** ^1^ Chongqing Key Laboratory for Oral Diseases and Biomedical Sciences, Stomatological Hospital of Chongqing Medical University, Chongqing, China; ^2^ Chongqing Municipal Key Laboratory of Oral Biomedical Engineering of Higher Education, Stomatological Hospital of Chongqing Medical University, Chongqing, China

**Keywords:** fibromodulin, SLRPs, inflammatory diseases, inflammation, immune response

## Abstract

Inflammation is an immune response that the host organism eliminates threats from foreign objects or endogenous signals. It plays a key role in the progression, prognosis as well as therapy of diseases. Chronic inflammatory diseases have been regarded as the main cause of death worldwide at present, which greatly affect a vast number of individuals, producing economic and social burdens. Thus, developing drugs targeting inflammation has become necessary and attractive in the world. Currently, accumulating evidence suggests that small leucine-rich proteoglycans (SLRPs) exhibit essential roles in various inflammatory responses by acting as an anti-inflammatory or pro-inflammatory role in different scenarios of diseases. Of particular interest was a well-studied member, termed fibromodulin (FMOD), which has been largely explored in the role of inflammatory responses in inflammatory-related diseases. In this review, particular focus is given to the role of FMOD in inflammatory response including the relationship of FMOD with the complement system and immune cells, as well as the role of FMOD in the diseases associated with inflammation, such as skin wounding healing, osteoarthritis (OA), tendinopathy, atherosclerosis, and heart failure (HF). By conducting this review, we intend to gain insight into the role of FMOD in inflammation, which may open the way for the development of new anti-inflammation drugs in the scenarios of different inflammatory-related diseases.

## 1 Introduction

Inflammation is a tightly regulated immune response that an organism defends itself against invading pathogens and responds to tissue injury under aseptic or sterile conditions. Pathogen-associated molecular patterns (PAMPs), expressed by foreign intruders such as viruses and bacteria, are recognized by pattern recognition receptors (PRRs), leading to an inflammatory response activation (1). In contrast, the inflammatory responses without pathogen infection are termed sterile inflammation, while tissue damage leads to the release of endogenous molecules, including intra- or extracellular origin, which are referred to as damage-associated molecular patterns (DAMPs) (2, 3). Both PAMPs and DAMPs could initiate immune responses by activating classical PRRs, including Toll-like receptors (TLRs) and multiple germ-line-encoded receptors (2, 4). Moreover, DAMPs can be sensed by non-PRR DAMP receptors, such as the receptor of advanced glycation end products (RAGE), triggering receptors expressed on myeloid cells (TREMs), several G-protein-coupled receptors (GPCRs), and ion channels (2). Traditionally, the process of inflammation can be divided into three phases, including inflammation, normal resolution, and post-resolution (5). If the process is uncontrolled, it will result in organ pathology or chronic inflammation including cancers, osteoarthritis, and cardiovascular (6–8). Indeed, chronic inflammatory diseases have been the major cause of death in almost all countries, which greatly affects a large group of the quality of life of individuals, creating a substantial burden on society, psychology, and the economy (9–11). Consequently, developing more effective and specific drugs targeting inflammation and resolution of inflammation in various inflammatory diseases has become an urgent demand for doctors and scientists.

Small Leucine-Rich Proteoglycans (SLRPs) belong to a diverse family of proteoglycans that are one of the major components of the extracellular matrix (ECM) and ubiquitously distributed in connective tissues, which are involved in the matrix organization and regulation of various cell growth and signaling (12–16). It is becoming increasingly clear that the SLRPs play critical roles in inflammatory responses by exhibiting pro-inflammatory or anti-inflammatory effects, as well as participating in the resolution of inflammation (17–20). For instance, biglycan-CD44 interaction could induce autophagy of M1 macrophages, thus increasing anti-inflammatory M2 macrophage depositions to influence the inflammatory response in the kidney (21). Additionally, in the cornea, lumican stimulated the recruitment of macrophages and polymorphonuclear neutrophils (PMN), accompanied by the elevated production of the pro-inflammatory cytokines tumor necrosis factor-α (TNF-α) and Interleukin-1β (IL-1β), which finally triggered the inflammation and corneal injuries healing (22). Similarly, another important SLRPs member, FMOD, has been shown to play a significant role during the inflammatory stage of diseases, such as the alteration of inflammatory cell infiltration during skin wound healing, and affecting the macrophage content and function in atherosclerotic (23, 24).

In this review, we mainly focused on the role of FMOD in inflammatory responses and diseases associated with inflammation, which may deepen our knowledge of inflammation during various inflammatory diseases and provide a new direction for anti-inflammatory therapies.

## 2 The molecular and structural hallmarks of FMOD

SLRPs are a large family of extracellular proteoglycans characterized by a core protein consisting of homologous amino acid residues and variable glycosaminoglycan (GAG) chains attached to core proteins that is often larger than a trisaccharide. SLRPs can be classified into five classes based on sequence homology at both the protein and genomic level, as well as the chromosomal organization (12, 14). FMOD belongs to the class II and four asparagine residues in its core protein could serve as acceptor sequences that can be attached with N-linked keratan sulfate chains (25, 26), which was originally isolated from bovine articular cartilage containing 376 amino acid residues with a core molecular mass of 42 kDa and flanked on both sides by domains lacking the repeat structure (25). FMOD was expressed in a variety of connective tissues such as tendons, sclera, serum, skin, etc (27). The name of FMOD was derived from its function to bind to the fibrillar types I and II collagens, which results in delayed and reduced collagen fibrils and modulates the mechanical properties of these fibrils (28). As a secreted protein, it can be secreted by various cells, such as fibroblasts, chondrocytes, keratinocytes, and melanocytes (29–33).

**Figure 1:**
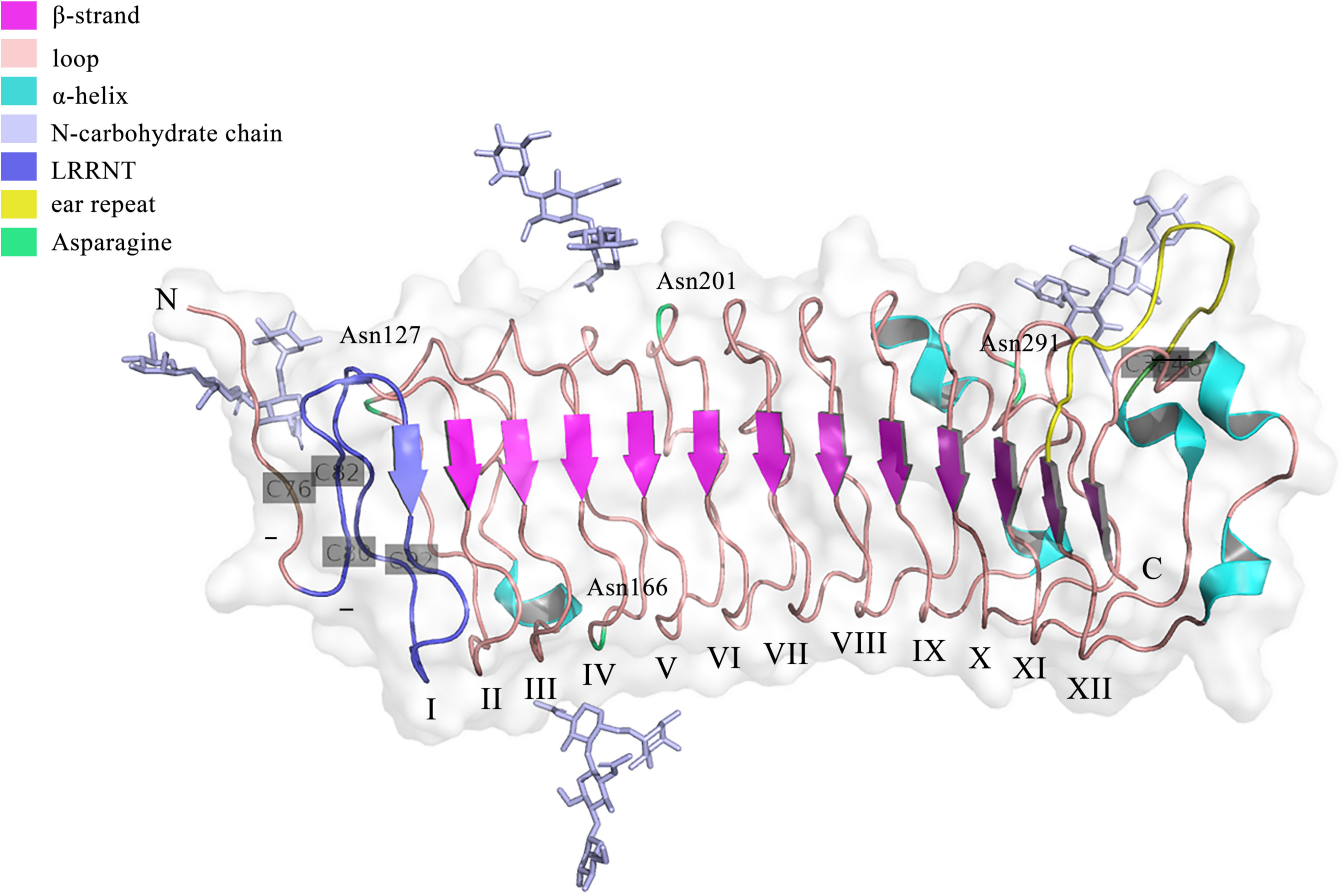
The crystal structure of the FMOD with lacking the disordered N-terminal structure. The structure was retrieved from Protein Data Bank (PDB), ID: 5mx0. FMOD displays typical SLRP structure features with 11 LRR motifs following LRRNT (also could be called LRR I). Three disulfide bonds locate at Cys76-Cys82, Cys80-Cys92, and Cys334-Cys367. Four potential N-linked glycosylation sites lie on the Asn127, Asn166, Asn201, and Asn291.

Genetically, *FMOD* is localized at chromosome 1 (1q32) in humans, which has a syntenic region in mouse chromosome 1 (34). The encoded regions of FMOD are composed of three exons, with a major central exon encoding nearly all LRRs, which shares overall homology of 90% in bovine FMOD (35). Previous studies have shown that sequence variations in the *FMOD* gene may be related to the pathogenesis of high myopia in humans (36). Structurally, FMOD protein can be divided into three main domains, including the N-terminal, C-terminal, and central domains. Except for the first 18 amino acids of a signal peptide that would be cleaved after protein secretion, the remaining N-terminal domain is the least conserved, which contains a tertiary structure with two loops formed by disulfide bridges between the four cysteine residues (25). The C-terminal domain is less conserved, which has a single loop containing two cysteine residues forming an intrachain disulfide bond (25). The central domain is the leucine-rich repeat (LRR) region with repeats of 20–30 amino acids with high homologous to decorin (25, 37). Besides, FMOD undergoes several post-translational modifications. For example, nine of ten N-terminal tyrosines are *O*-sulfated (38), and Asn127, Asn166, Asn201, and Asn291 are N-linked glycosylated with four sugar molecules (NAG-NAG-BMA-FUC) observed in each glycan chain at the electron density, while whether these glycans would be extended by keratan sulfate could not be determined from the electron density (39), which influence the half-life of proteins secreted into circulation and enhance the rate of secretion of proteins from cells (40). The crystal structure of FMOD was first obtained in 2017 (39). Like other LRR proteins, FMOD shows several hallmarks of the curved solenoid structure: 1) the N-terminal cap (LRRNT, 79-106) contains two conserved disulfide bonds (Cys76-Cys82, Cys80-Cys92) and seals the hydrophobic core of the LRR region, which is also associated with the strand to the curved parallel β-sheet that dominates the concave face of FMOD (39); 2) Following LRRNT, there are 11 LRRs with 20 to 27 residues in FMOD, which share the consensus sequence LxxLxLxxNxL and display a long-long-short pattern, as previously seen in decorin (39); 3) Residues 3–6 of each LRR form one β-strand to the concave face of the curved solenoid and the convex face consists of loops and turns (37); 4) FMOD also show the characteristic of SLRP classes I, II or III, termed ‘ear repeat’, which spans from Cys334 in LRR XI to the β-strand of LRR XII (39) (**Figure 1**). Interestingly, unlike that class I SLRPs, such as decorin, forms stable dimers, FMOD was mainly in monomeric in physiological solution (37). However, the current crystal structure of FMOD did not contain the disordered N-terminal structure, which displays a pivotal role in the biological function of FMOD, such as binding to the collagen (39, 41). It seems that the disordered N-terminal structure of FMOD is flexible and variable based on its specific function, which needs to be further confirmed.

Functionally, FMOD has been shown to interact with collagen and regulate collagen fibril growth, both *in vivo* and *in vitro* (28). Previous studies have shown that it is bound to collagen fibers through three proven binding sites, two of which were in the leucine-rich repeat core structural domain located at LRR7 and LRR11 (42–44), which inhibited the fibril formation rate (45). The remaining one was in the tyrosine sulfate domain links to the N-terminal, which has a high affinity for binding to collagen, contributing to shortening the fibrinogenesis lag phase and influencing the arrangement of collagen molecules in the early fibrillogenesis stage (45). Additionally, FMOD was shown to form a complex with lysyl oxidase to increase its activity and influence its site-specific cross-linking with collagen (46, 47). Since inflammation often results in the degradation of collagen in the ECM and improving ECM may be a potential therapeutic strategy for inflammation disease (48–50), whether FMOD could be a therapeutic agent for anti-inflammation via regulating collagen fibrillogenesis remains elusive and needs to be further studied. Furthermore, FMOD shows the ability to bind to mammal transforming growth factor β (TGF-β) isoforms with two binding sites and could slightly bind to latent recombinant TGF-β1 (51). Considering that TGF-β exerts multi-faceted effects in inflammation based on various cellular and environmental contexts, whether FMOD could regulate inflammation in various inflammatory diseases via regulating TGF-β signaling remains unclear. Also, the specific binding mode between FMOD and TGF-β, as well as the molecular mechanism of FMOD regulating TGF-β signaling in inflammation need to be largely explored. Interestingly, FMOD was shown to bind to the complement element C1q, which is important for the regulation of inflammation, and it will be detailly discussed in the following part.

## 3 FMOD in inflammatory response

### 3.1 FMOD and the complement system

The complement system, comprising the soluble and cell membrane proteins, plays a crucial role in the innate immune response to defend against pathogens and maintain host homeostasis (52). Complement proteins act as a recognizer and transmitter of exogenous and endogenous related danger signals during immune responses (53, 54), which help microbial recognition and initiation of phagocytosis and inflammation by activating the classical, alternative, and lectin pathways (55). The activation of complement can be divided into four main steps, including initiation of complement activation (classical, lectin, or alternative), C3 convertases activation and amplification in which C3 is cleaved to C3a (pro-inflammation) and, C3b (eliminating microorganisms in a non-inflammatory manner), C5 convertases activation in which C5 is cleaved to C5a (pro-inflammation) and C5b (initiating the terminal pathway), and the membrane attack complex (MAC; C5b–9) formation, finally resulting in cytolysis or cell activation. These activation fragments bind to their respective complement receptors to cause various biological responses, including phagocytosis, immune adherence and removal, cell migration, tissue regeneration, cell activation, and modulation of pattern recognition receptor-induced responses (52, 55–57). Notably, the complement system is tightly regulated by proteins such as factor H (FH) and factor I (FI) to decay the C3 convertases or mediate the cleavage of activation fragments (52, 58), thereby avoiding unexpected complement overactivation. Previous studies have identified that the complement system plays a key role in the inflammation of various diseases. For example, targeting C3a-C3aR/C5a-C5aR axis could control maladaptive immune-inflammatory consequences of the complement pathways in severe coronavirus infectious disease 2019 (COVID-19) (59). Moreover, a previous study showed that MAC-induced chondrocytes produced more inflammatory and degradative molecules, which were colocalized with matrix metalloprotease 13 (MMP-13) and activated extracellular signal-regulated kinase (ERK) around chondrocytes in human osteoarthritic cartilage, which plays a critical role in OA synovial fluid in the pathogenesis of osteoarthritis (60).

**Figure 2:**
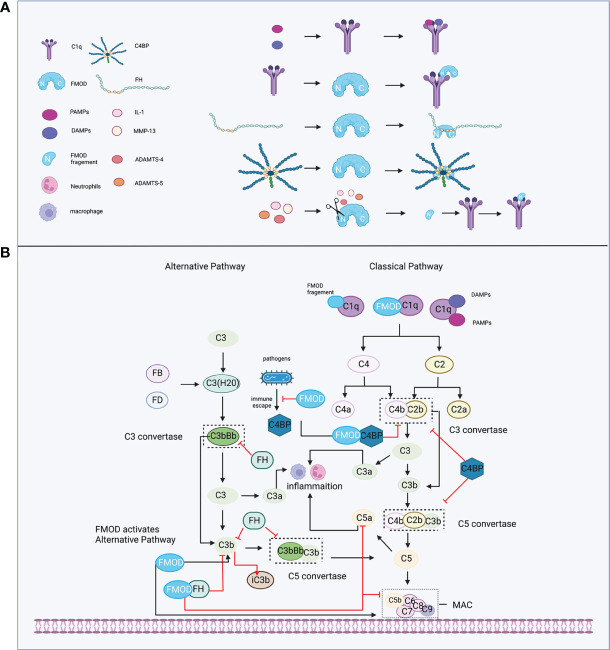
FMOD and the complement system. **(A)** C1q recognizes immune complexes or FMOD and binds with them; complement inhibitor proteins FH and C4BP bind with FMOD; FMOD could be cleaved by MMP-13, IL-1, ADAMTS-4, ADAMTS-5, and its fragment binds with C1q. **(B)** FMOD binds to C1q resulting in the deposition of C4b and C3b to activate the classical pathway; FMOD activates the alternative pathway; FH acts as a cofactor for FI to prevent the formation of the C3 and C5 convertases and acceleration of C3b decay; C4BP acts as a cofactor for FI to prevent the formation of the classical C3 and C5 convertases; FMOD binds with C4BP to avoid the immune evasion of the pathogen and not influence the C4bP function; FMOD binds with FH to inhibit C5a and C9 depositions but increase iC3b. Created with BioRender.com.

During the initiation of the complement system, the classical pathway can be activated in either an immune complex-dependent or independent manner, while the alternative pathway is in a constant state of low-level activation (“Tick-over”), allowing for immediate response upon microbial challenge (61). Tick-over is the spontaneous hydrolysis of a labile thioester bond, which converts C3 to a bioactive form of C3(H_2_O) in the fluid phase. Then, C3(H_2_O) is bound to factor B (FB) (62) and cleaved by factor D (FD) to form a fluid phase C3 convertases complex, termed C3(H_2_O)Bb, which could interact and cleave native C3 molecules to C3a and C3b (61). FMOD was shown to hold the ability to participate in both the classical and alternative pathways of complement to regulate the inflammatory response (**Figure 2**). For example, FMOD can cause the deposition of C1q, which is a recognition molecule of the classical pathway and is mainly produced by immature dendritic cells, monocytes, and macrophages (63) to recognize immune complexes or other structures. The N-terminal of FMOD can directly bind to the globular heads of C1q to activate C1, which subsequently results in the deposition of C4b and C3b to initiate the early steps of the classical pathway (64). Moreover, C3b and C9 deposition were also observed in C1q-deficient serum and factor B-deficient serum with FMOD administration, which suggests that FMOD could also activate the alternative complement pathway (64). In the specific disease setting, the N-terminal fragment of FMOD was cleaved by inflammatory cytokine or matrix metalloproteinases (MMPs) and these cleaved fragments could bind with C1q, leading to the activation of classical pathways to aggravate the inflammatory response of osteoarthritis (discussed below) (65–67).

On the other hand, FMOD was found to bind with FH at the sixth to eighth complement control protein (CCP) domain of FH (68), while the deglycosylated FMOD showed a higher affinity to bind to FH than the keratan sulfate-containing form, which suggests that the binding site for FH on FMOD is localized to the polypeptide chain and the keratan sulfate causes steric hindrance for the interaction (64, 68). Interestingly, the binding of FMOD with FH further inhibited the alternative pathway, causing the lower release of C5a to directly repress neutrophil adhesion on the joint endothelium igniting inflammation (69, 70). Increased iC3b release that can bind with complement receptors to mediate phagocytosis and inhibit alternative pathway activation (71), and lower deposition of C9 to prevent excessive host cell damage (64). Moreover, the FH-related (FHR) proteins like FHR1 and FHR5 could compete with FH for binding to C3b, and support the assembly of the alternative pathway C3 convertases, thereby enhancing alternative pathway activation (72, 73). Interestingly, FHR proteins could disturb the binding between FMOD and FH, which resulted in the decreased FH cofactor activity and the increased deposition of C3-fragments, factor B and C5b-9 to enhance local complement activation and promote inflammation under pathological conditions (74). Additionally, FMOD was shown to bind to C4b binding protein (C4BP) at the central core including CCP8, which is a complement inhibitor that represses the formation of the classical pathway C3 and C5 convertases (61, 75), as well as serves as a cofactor for FI, participating in the proteolytic degradation of C4b and C3b (76), which does not inhibit the activity of C4BP (77). However, deleting C4BP from serum significantly enhanced complement activation initiated by FMOD with higher deposition of C9 (77). Notably, C1q, FH, or C4BP binds to FMOD at different binding sites, and FMOD is capable of binding and activating C1q, as well as simultaneously binding to FH and C4BP (64, 77). Several pathogens like *Moraxella catarrhalis*, which caused acute otitis media in children, as well as stimulated acute exacerbations in chronic obstructive pulmonary disease patients, could recruit C4BP from the host to immune evasion (78–80). A recent study revealed that FMOD could competitively bind to C4BP to inhibit its binding to the surface of *Moraxella catarrhalis*, resulting in increased C3b/iC3b deposition, MAC formation, and subsequently avoided the immune evasion of the pathogen and decreased survival of bacterial (81).

Collectively, the multifaced effects of FMOD on the complement system may suggest its critical role in the delicate regulation of complement activation under physiological conditions. When the balance is destroyed in the pathological state, FMOD may exert pro or anti-inflammatory effects in the different scenarios of diseases, and the interaction of FMOD and complement may prevent the immune evasion of the pathogen, which is worth being deeply explored in the future.

### 3.2 FMOD and immune cell

The acute inflammatory response is a complex but highly coordinated process involving molecular, cellular, and physiological alterations (5). After an injury, the necrotic cells initiate DAMPs and PAMPs which are recognized by innate receptors on tissue-resident cells to produce various inflammatory mediators such as complement, chemokines, and cytokines, then recruiting leukocytes including neutrophils, macrophages, and dendritic cells (DCs) to trigger inflammatory responses at the injury site (5, 82).

Within 24 hours of the injury, neutrophils are quickly attracted to the wound site and fight off invasive pathogens through a variety of antimicrobial reactions, such as phagocytosis, toxic granules, oxidative burst, and neutrophil extracellular traps (83–85). However, the consistent existence of neutrophils at the injury site was detrimental to proper tissue repair by secreting proteases including MMPs to degrade ECM components, resulting in excessive inflammatory responses, finally causing impaired healing and chronic wound formation (86).

After 48-72 hours, the number of macrophages reaches a peak at the injury site (87), which plays critical roles in pro-inflammation, resolution of inflammation, and tissue reorganization (88). Naive macrophages can be differentiated in response to external stimulation to M1 or M2 macrophages, each of which has distinct functions that M1- phenotype macrophages induce pro-inflammatory cytokines and chemokines to eliminate pathogens, while the M2-phenotype macrophages diminish the inflammatory response, and promote tissue repair and healing by producing anti-inflammatory molecules, collagen, and elastin precursors (89, 90). Recently, FMOD has been shown to involve in macrophage differentiation and polarization via regulating the TGFβ signal pathway. FMOD was found to be cleaved by MMP-8 to increase the bioavailability of TGF-β1 to induce the M2-phenotype macrophages differentiation and polarization (91). Furthermore, the expression and activation of FMOD could be regulated by Notch1 haploinsufficiency, which repressed the expression of TGF-β2 to reduce the expression of various genes associated with M2 polarization (92). Moreover, FMOD was associated with macrophage content and function in the apolipoprotein E (ApoE)/FMOD-null mice with atherosclerosis, which influences the content of macrophage in different areas and the ability of macrophage uptaking lipid or secreting anti-inflammation factors in plaque (discussed below) (93).

With cytokine or chemokine production, monocyte precursors upon infection or injury differentiate into dendritic cells (DCs) which are professional antigen-presenting cells (APCs) that are critical for the initiation of immune response and act as a bridge between innate and adaptive immunity (94). Plasmacytoid DCs (pDCs), a subset of DCs, has been shown to orchestrate the beneficial immunoregulatory interaction among commensal microbial molecules to regulate T cell to produce IL-10 through innate and adaptive immune (95). A recent study found that the absence of FMOD led to the increases of pDCs to produce more type I IFN and activate the effector T cells, which induced a stronger inflammatory response and destroyed the epithelial barrier in a mouse model of dextran sodium sulfate (DSS) -induced acute colitis (96). Additionally, FMOD has been found to affect the function of immune cells to influence inflammatory reactions in several autoimmune diseases like systemic lupus erythematosus. In the study of induced lupus erythematosus in mice with different pigments, compared with white *fmod* ^+/+^ mice, the black *fmod* ^+/+^ produced low levels of FMOD, while white *fmod* ^-/-^ mice developed more severe inflammation accompanied by increased numbers of DCs (97), which indicate that FMOD may be involved in the inflammation of lupus erythematosus by regulating the expansion of inflammatory DCs.

Together, the above results indicate that FMOD may affect the functions of multiple immune cells such as DCs and macrophages during inflammation in various diseases. However, whether and how FMOD is engaged in the regulation of other immune cells’ activities remains unclear. On the other hand, it seems that the hallmark of FMOD regulating TGFβ1 signaling is mostly the key factor that decides the effects of FMOD on the activities of the immune cells during inflammatory responses, while the specific mechanisms need to be elucidated in various immune cells and inflammatory processes in the future.

## 4 FMOD and diseases associated with inflammation

### 4.1 FMOD and skin wound healing

Cutaneous wound healing is a complex process comprising various immune and structural cells, which secret various cytokines, chemokines, and growth factors to orchestrate the phases of healing (98). The classical physiological process of wound healing can be divided into four steps: hemostasis, inflammation, proliferation, and remolding (99). In normal skin wound healing, the inflammation responses usually continue for 2–5 days and gradually resolve once the harmful stimuli have been removed (100). However, over-inflammation always leads to a persistent inflammation phase and delaying wound healing, finally resulting in chronic inflammation wounds or hypertrophic scars formation (101). The over-inflammation is accompanied by the prolonged infiltration of pro-inflammatory immune cells such as neutrophils and monocytes, as well as the failed phenotypic conversion of macrophage from a proinflammatory to an anti-inflammatory (98, 99). Besides, the ability of macrophages to clear dead neutrophils is limited, thus producing many inflammatory mediators to trigger inflammation responses (102). Therefore, adjusting the polarization of macrophages, inhibiting pro-inflammatory factors, and anti-inflammatory cytokines treatment, have been thought to be promising therapies for skin wound management (98).

Although FMOD knock-out mice did not exhibit apparent defects in the skin (103), a wider distribution of collagen fibril diameters and enlarged interfibrillar spaces between collagen fibrils was observed (104). Importantly, previous studies have identified FMOD as a biologically significant mediator of skin wound healing, including regulating various fibroblast activities, angiogenesis, and inflammation, which is associated with scarless skin wound healing and skin fibrosis (15, 105–109). For example, loss of FMOD delayed skin wound healing, and led to scar formation in the fetal wounds, while restoration of FMOD could rescue the phenotype with scarless wound healing (107). Furthermore, FMOD acts as an activity regulator precisely regulating TGFβ activity to elicit fetal-like cellular and molecular phenotypes during adult skin wound healing (109). Furthermore, recombinant FMOD administration could promote cutaneous wound healing in various preclinical animal models, such as mice, rats, and pigs (15). Excitedly, a synthesized FMOD-derived peptide did not raise any safety concerns in a human phase I clinical trial, and it is now being tested in a phase II clinical trial for managing skin wound healing (Clinicaltrials.gov: NCT03880058).

FMOD may have the biological effect of alleviating inflammatory responses in the process of skin wound healing. Previous studies showed that the wound healing with FMOD-null mice exhibited delayed wound closure and increased scar size (23, 110). Notably, During the inflammatory stage of wound healing, FMOD-null mice exhibited more inflammatory cells in the wounds, including neutrophils, monocytes, and macrophages, which were characterized by the earlier appearance of macrophages after injury. Moreover, the level of type I TGF-β receptor was increased in the single inflammatory cell during the inflammatory phase and FMOD-null wounds exhibited higher sensitivity to TGF-β (23). Furthermore, the increased TGFβ1 signals were detected in migrating epidermis and granulation tissue of FMOD-null wounds at the early stage, which is consistent with the appearance of macrophages, while the Peak TGFβ1 expression of ECM was associated with fibroblasts density rather than inflammatory cells in FMOD-null wounds (23). Importantly, macrophages have been shown to induce granulation tissue and myofibroblast differentiation during the early stage of the repair response, and early-stage macrophage depletion significantly reduced granulation tissue formation, impaired epithelialization, and scar formation (111), which suggests FMOD may regulate macrophages function to influence the progress of inflammation stage and the transition from inflammation to reconstruction during skin wound healing. On the other hand, TGF-β1 was mainly secreted by macrophages during wound healing (112), while FMOD could regulate macrophage polarization *in vitro*, which was found to delicately regulate TGFβ activity to elicit fetal-like skin wound healing (109). Consequently, a tight relationship may exist among FMOD, macrophages, and TGFβ1 during skin wound healing, while the specific mechanism of how these elements collaborate or restrict each other to participate in the regulation of the inflammation process during skin wound healing needs to be further explored.

### 4.2 FMOD and osteoarthritis

Osteoarthritis (OA) is the most common form of arthritis which exhibits typical structural changes including cartilage degradation, subchondral bone remodeling, osteophyte formation, and changes in the synovium and joint capsule (113). With the continuous deepening of the understanding of OA, it has been regarded as an inflammatory disorder but not only a degenerative illness (114, 115). In OA, chondrocytes, synoviocytes, and synovial fibroblasts are the source of pro-inflammatory cytokines and matrix-degrading enzymes, while infrapatellar fat pad (IFP) is a site of inflammatory mediators to contain considerable amounts of immune cells such as macrophages and T cells (116), which causes a persistent inflammatory response to change the anatomical and physiological functions of the joint by affecting cell signaling pathways, gene expression, and joint tissue (117, 118). Currently, therapies for osteoarthritis are limited to symptom-relieving drugs and total knee arthroplasty for severe cases, while drugs targeting the underlying biological causes of osteoarthritis are not available in the market (117). Therefore, it is important to identify the development and progression of osteoarthritis, which may benefit further developing a novel OA therapy.

Recent studies have identified that FMOD plays a vital role in the progression of osteoarthritis. FMOD-null mice exhibited a higher incidence of osteoarthritis in knee joints, which occurred at 36 weeks in the articular cartilage, subchondral bone, ligaments, and menisci (119), while the ECM and type II fibrils were not altered in the articular cartilage (119). Since biglycan (BGN) and FMOD display a complementary role in various physiological and pathological processes, the mice with the absence of Bgn and Fmod successively developed gait impairment, ectopic tendon ossification, and severe premature osteoarthritis including cartilage degeneration and damage at an early stage (between 1 and 2 months), and its progression was very rapid (complete erosion of the articular cartilage between 3 and 6 months) (120). Notably, the collagen fibrils in the articular cartilage of the FMOD-deficient mice were also not different from those observed in the WT cartilage (120), which suggests that the osteoarthritis was not caused by collagen defects in the articular cartilage of FMOD null mice. A similar phenomenon was detected in the temporomandibular joint (TMJ) of Fmod and Bgn double-knockout mice, which developed OA in the TMJ after 6 months and TMJ was extremely destroyed after 18 months (121). Interestingly, the cell proliferation observed in chondrocyte clusters was increased at an early stage. However, cell proliferation was decreased at the onset of OA (121). In addition, in *bgn* ^-/-^; *fmod* ^-/-^ mandibular condylar chondrocyte(MCCs), TGF-β1 signal transduction was increased to enhance chondrogenesis, collagen II and aggrecan at an early age (122). With aging, the overactive TGFβ1 increases MMPs and degrades ECM, resulting in collagen II and cartilage degradation (122). This suggests a more delicate and complicated regulatory network that FMOD is involved in during the pathogenesis of OA, and there needs more research to identify the relationship between FMOD, TGFβ1 and chondrocytes during the progression of OA.

Additionally, FMOD was thought to act as a barrier preventing cell adhesion and protecting the joint. Neutrophil has been shown to secret pro-inflammatory mediators and elastase, which regulates the transport of other leukocyte subsets, increases the collagen II binding with the antibody on the articular cartilage, and cleaves cartilage surface matrix including FMOD to promote cartilage damage (115, 123, 124). On the contrary, the complete FMOD can inhibit polymorphous-clear neutrophil (PMN) adhesion (125), and partially reconstruct the surface of elastase-digested cartilage to prevent sustainable cartilage damage (126).

Moreover, the fragmentation of FMOD may affect the ability of its binding with collagen and active complement which are both involved in the progression of osteoarthritis. The MMPs have been shown to participate in the balance between anabolism and catabolism in articular cartilage (127), while MMP-13 is the key enzyme in the cleavage of collagen II and plays an essential role in cartilage destruction in OA (128). It has been demonstrated that MMPs including MMP-13, a disintegrin and metalloproteinase with thrombospondin motif-4 (ADAMTS-4), and ADAMTS-5 can cleave FMOD (67), and the cleaved N-terminus of FMOD is similar to the fragment obtained by interleukin-1 (IL-1) treatment (65, 66). However, the purified intact FMOD could not be cleaved by IL-1 and MMP-13 (65). This suggests that cleavage of fibromodulin occurred at the site where the molecule is bound to collagen fibrils, while following FMOD cleaved, the fibrillar network was altered to lead to the exposure of sites which was subsequently confirmed by the cleaved type II collagen fibers (65, 66).On the other hand, complements have been found in significantly increased abundance in OA synovial fluid (129) and play a key role in OA synovial fluid in the pathogenesis of osteoarthritis (60). Notably, the pulverized osteoarthritic cartilage from human has higher expression of FMOD and higher deposition of MAC, while FMOD was present at higher concentrations in osteoarthritic compared to healthy synovial fluid (60). In addition, sublytic MAC increased the multiple gene expression of chondrocytes implicated in osteoarthritis including MMPs, ADAMTSs, and inflammatory cytokines, and it was colocalized with MMP-13 and activated extracellular signal-regulated kinase (ERK) (60). However, at present, there is no further research to explore the relationship among FMOD, chondrocytes, MAC, and MMPs in OA. Furthermore, The site of FMOD binding with C1q can be competitively bound to the NC4 domain of cartilage-specific collagen type IX collagen to inhibit complement activation to reduce the degradation of collagen II and the excessive inflammatory response (130). Since FMOD can directly bind with C1q, FH, and C4BP at different binding sites to regulate the complement system, whether FMOD could regulate the progression of OA by mediating complement system needs to be further elucidated.

In conclusion, the FMOD and its fragment may exhibit multiple functions such as collagen binding, complement activation, and TGF-β1 signaling regulation to influence the progression of OA. However, more research is needed to explore the specific mechanism of FMOD and its fragments in the regulation of inflammation during OA progression, especially the specific relationship among FMOD, collagen II, and MMPs in OA, which may pave the way to develop an FMOD-related agent for OA management or a new diagnostic biomarker in the progress of osteoarthritis.

### 4.3 FMOD and tendinopathy

Tendon is composed of highly arranged collagen fibers and other ECM components, including oligomeric matrix protein, elastin fibrils, and SLRPs (131). Once tendon injury occurs, the natural healing ability of tendons is rather limited due to hypocellularity and hypovascularity (132). Similar to skin wound healing, tendon healing processes can be divided into three phases, including inflammation, proliferation, and remodeling phases (133). The inflammation in tendinopathy contains three distinct cellular compartments (stromal, immune-sensing, and infiltrating compartments), each contributing to a complex condition of inflammatory responses affecting tendon homeostasis (134, 135). The failed resolution of an inflammatory response in tendons leads to chronic persistent disease that is one of the hallmarks of tendinopathy (136). Thus, the balance of immune-stromal and cell-matrix interactions may play an important role in the resolution of tendon inflammation (134).

FMOD, as a key regulator of tendon strength and fibril maturation, has been shown to participate in tendinopathy pathogenesis (137). FMOD deficiency mice exhibited an altered morphological phenotype in the tail tendon with fewer and abnormal collagen fiber bundles, despite it could be partly rescued by an increase of lumican (LUM) (138). Furthermore, compared with WT mice, the absence of Lum and Fmod led to extreme tendon weakness, while the deficiency of FMOD alone resulted in a significant reduction in tendon stiffness (139). Similar phenotypes were observed in the deficiency of BGN and FMOD mice, including alteration of collagen fibrils and weak tendons (120). Importantly, overexpression of FMOD could enhance tendon healing *in vivo* and *in vitro* (140). In addition, FMOD/gHA‐hydrogel was shown to significantly promote tendon healing histologically, mechanically, and functionally (141). However, the specific role of FMOD in the regulation of inflammation during tendon wound healing remains unclear.

Tendon stem/progenitor cells(TSPCs) were identified in 2007 (142). It has been shown to have the ability of multi-differentiation potency to differentiate into tenocytes as well as non-tenocytes, including adipocytes, chondrocytes, and osteocytes (143, 144). Besides, TSPCs participate in the regulation of inflammation during the healing of acute tendon injuries (145). TSPCs seeded in knitted silk-collagen sponge scaffolds promoted regeneration of the rotator cuff in a rabbit model by inducing tenogenic differentiation and secreting anti-inflammatory cytokines that prevented immunological rejection (146). Notably, FMOD and BGN are two critical components in the TSPCs niche, which controls the self-renewal and differentiation of TSPCs (142). Furthermore, In double -knockout of Bgn and Fmod mice, TSPCs proliferated faster, formed larger colonies, and formed bone-like tissues in addition to tendon-like tissues compared with wild-type mice which formed only tendon-like tissues. This difference may be mediated by modulating bone morphogenic protein (BMP) activity, which is a part of the large TGFb signaling pathway (142, 147, 148). A recent study found that in the early inflammatory process of tendon repair, the increased inflammatory cytokines, including IL-6, IL-10, and IL-1β that promoted the proliferation and migration of tendon cells, were observed accompanied by reduced FMOD and lumican expression, which resulted in the inhibition of TSPCs differentiation to influence the healing in the tendon (149–151), but whether the reduced FMOD and lumican expression are related to the excessive inflammatory responses needs to be further studied. Interestingly, both *in vitro* and *in vivo* studies showed that FMOD can improve tenocytes migration and collagen matrix organization by modulating TGFβ (51, 140) and connective tissue growth factor (CTGF), while a recent study has shown that CTGF-enriched CD146+ TPSCs reduced pro-inflammatory M1 macrophages in the early healing phase and expressed anti-inflammatory IL-10 and TIMP-3 via JNK/signal transducer and activator of transcription 3 (STAT3) signal (152). Whether FMOD participates in this process by regulating CTGF signaling during the inflammation phase in tendon healing needs to be further studied.

In conclusion, FMOD not only influences the ECM remodeling during tendon reconstruction by regulating the arrangement and maturation of fibers but also may influence the activity of TSPCs during the inflammatory phase of tendon healing, thereby affecting tendon injury healing. However, there needs to confirm the molecular mechanisms of FMOD in the inflammatory phase and the ECM remodeling phase during tendon wound healing, e.g. whether FMOD regulates TSPCs activity during the inflammatory phase through TGFβ1, thus allowing a better transition from the inflammatory phase to the tendon remodeling phase, finally promoting poor tendon healing.

### 4.4 FMOD and atherosclerosis

Atherosclerosis is a well-coordinated pathological process involving the entering and accumulation of atherogenic lipoproteins in the sub-endothelium and subsequent deposition of extracellular matrix, inflammatory cells, smooth muscle cells, necrotic cellular debris, and neo-vasculature with intraplaque hemorrhage, leading to the formation of an atherosclerotic plaque (153, 154). Advanced atherosclerotic plaque can encroach upon the arterial lumen and impede blood flow. Once the plaque disrupts and provokes the formation of a thrombus, it will occlude the lumen leading to tissue ischemia (155). Lipids and the inflammatory response play an important role in the initiation, progression, and destabilization of atherosclerotic plaques (154). Plaque rupture is generally attributed to collagen depletion in the fibrous cap of the plaque resulting from matrix degradation by MMPs, which are secreted by inflammatory cells, predominantly macrophages, in the plaque (156). Consequently, anti-inflammatory interventions have been thought to be able to forestall atherosclerotic complications. Particularly, the NLRP3 inflammasome as well as the downstream cytokines IL-1β, IL-18, and IL-6 are attractive candidate targets to be interfered with (8). However, interfering with the inflammatory pathways may also damage the host defenses. Due to this fact, promoting the resolution of inflammation without impairing defenses may be one possible avenue, such as reducing leukocyte infiltration and the production of pro-inflammatory mediators, or increasing the containment and phagocytosis of cellular debris and apoptotic cells (8).

Recently, FMOD has been found to act as a risk factor for atherosclerosis. FMOD expression was upregulated and localized at the area of macrophage-like cells in atherosclerotic aortas of ApoE/LDL deficient mice (157). Moreover, FMOD was detected in carotid atherosclerotic plaques from symptomatic and asymptomatic patients, while FMOD expression was significantly higher in plaques obtained from patients with diabetes and in those with an increased incidence of postoperative neurological events (24). Furthermore, the expression of FMOD was found to be positively correlated with the expression of the pro-inflammatory cytokines including macrophage inflammatory protein 1β (MIP-1β) and soluble CD40 Ligand (s-CD40L), as well as the expression of vascular endothelial growth factor (VEGF), while FMOD expression had an inverse association with IL-10 expression in human plaques (24). Moreover, in ApoE-null/FMOD-null mice, plaque size was decreased accompanied by reduced lipid accumulation and macrophage content in plaque (93), while the RAW264.7 macrophages have less lipid accumulation, as well as increased IL-6 and IL-10 production on FMOD-deficient ECM (93). Notably, the proliferation of smooth muscle cells (SMCs) and macrophages were detected in low and oscillatory shear stress carotid lesions (93), while the SMCs could contribute to arterial inflammation by being transformed into macrophage-like or fibroblast-like cells in plaque (8). Consequently, it is indicated that FMOD may influence atherosclerotic plaque development by regulating the function of macrophages and SMCs, which suggests that FMOD may be a potential target for atherosclerosis treatment. However, more research is needed to further confirm the role of FMOD in the regulation of inflammation during atherosclerosis development.

### 4.5 FMOD and heart failure

Heart failure (HF) is a disorder associated with low-grade immune activation and inflammation, which progresses accompanied by pressure overload of the heart to lead to cardiac remodeling (158). Cardiac remodeling comprises cardiomyocyte hypertrophic growth, extracellular matrix alterations, and inflammatory responses (159). After myocardium injury, the inflammatory response induced by the innate immune system upregulates a portfolio of cytoprotective responses that provide the heart with a short-term adaptation to increased stress (physiological inflammation) (160, 161). Pro-inflammatory cytokines (such as IL-1β and IL-6), chemokines, DAMPs, complement systems, and immune cells such as macrophages play important roles in this process (162, 163). Once the inflammatory response becomes dysregulated and results in chronic inflammation, it will lead to left ventricular (LV) dysfunction and LV remodeling, gradually causing HF (164). Currently, approaches targeting inflammation in patients with HF can be broadly divided into targeted anti-cytokine therapies, anti-inflammatory therapies, immunomodulatory therapies, and strategies targeting autoimmune responses (165).

However, the poor understanding of the biology of persistent inflammation during chronic LV remodeling emphasizes the compelling need to conduct advanced translational efforts in this field to benefit the development of biological drugs (164). Emerging evidence shows that inflammation is essential in HF pathogenesis and leads to ECM remolding, in which FMOD may play a critical role. the chemokine CXCL13 was found to be involved in cardiac remodeling during HF via activating CXCR5 on myocardial fibroblasts and inducing the expression of SLRPs through the ERK1/2 pathway (166). In the CXCR5 knockout mice, a CXCL13 receptor (167), the mortality and severe left ventricles dilatation were increased during a follow-up of 80 days after aortic banding accompanied by decreased SLRPs expression, including FMOD and lumican (166). Besides, in clinical and experimental heart failure studies, compared with normal patients and mice, FMOD expression was 3-10-fold upregulated in the hearts of heart failure patients and mice, while increased FMOD expression was found in both cardiomyocytes and cardiac fibroblasts by NF-κB stimulation (168). Moreover, upon aortic banding, the left ventricles of FMOD-KO mice altered the infiltration of leukocytes, which may lead to increased cardiomyocyte size and hypertrophic phenotype (168).

In conclusion, previous studies have suggested that FMOD may play an important role in the inflammatory response and ECM remodeling during heart failure. Accordingly, it may be a potential biomarker as L. Adamo (164) suggested that more biomarkers were used to identify a population of patients with heart failure who would benefit from targeted anti-inflammatory strategies. However, the specific role of FMOD in the inflammation of heart failure needs to be further clarified.

## 5 Conclusion

FMOD has been extensively studied to participate in a variety of disease processes including angiogenesis, fibrosis, and tumors. In this review, we focus on the role of FMOD in inflammatory responses and diseases associated with inflammation, which aims to convince the reader that FMOD not only exhibits expression variations in the inflammatory response of diseases but more importantly, it plays a causal or maker role in the regulation of inflammation during various disease processes (**Table 1**). For example, it can activate inner immunity by binding with the complement system. In addition, it may influence the activity of immune cells such as macrophages by interacting with the TGFβ signal pathway during the progression of diseases like wound healing. Moreover, it may be a biomarker in the chronic inflammation of heart failure. Current studies strongly suggest that FMOD exhibits the potential ability as the treatment agent for skin or tendon wound healing, and OA management, However, there still lacking the deeply explored studies on the specific role of FMOD in other inflammation-associated diseases, such as atherosclerosis. Also, the specific mechanisms of FMOD involved in the regulation of inflammation during the pathogenesis of these diseases need to be further clarified, such as whether it influences the activity of cells in the inflammatory phase to affect extracellular matrix reconstitution in skin wound and tendon healing, which may benefit doctors and scientists to develop a new generation of therapeutic strategies for inflammatory diseases.

**Table 1:** The role of FMOD in the various inflammatory diseases.

Disease	Model	Biological effect	References
Skin wound healing	*fmod-/-* mice	delayed wound closure and increased scar size; Elevated inflammatory cell infiltration including macrophages	(23, 110)
Osteoarthritis	*fmod -/-* mice	a higher incidence of osteoarthritis in knee joints	(119)
	*bgn-/-;fmod-/- mice*	gait impairment, ectopic tendon ossification, and severe premature osteoarthritis; Accelerated OA onset and development; Influenced the proliferation of MCCs and ECM by TGFβ1 in TMJ	(120–122)
	Bovine cartilage slices	FMOD inhibited polymorphic clear neutrophil (PMN) adhesion;	(125)
	Human samples	The higher expression of FMOD was accompanied by increased deposition of MAC in pulverized osteoarthritic cartilage; FMOD was present at higher concentrations in osteoarthritic compared to healthy synovial fluid.	(60)
Tendinopathy	Tendon wound healing mice	FMOD enhanced tendon healing *in vivo* and *in vitro*	(140)
	Tendon-derived progenitor cells	The lower expression of FMOD was accompanied by increased inflammatory cytokines, including IL-6, IL-10, and IL-1β; inhibited tendon-derived stem cells(TDSCs) differentiation.	(149–151)
Atherosclerosis	*In ApoE/LDL-null mice*	FMOD expression was upregulated and localized at the area of macrophage-like cells	(157)
	Human samples	Increased postoperative neurological events; Increased expression of the pro-inflammatory cytokines, including MIP-1β, s-CD40L, and VEGF; decreased IL-10 production.	(24)
	In *ApoE-null/FMOD-null* mice	Decreased plaque accompanied by reduced lipid accumulation and macrophage content; less lipid accumulation, as well as increased IL-6 and IL-10 production; Increased SMCs and macrophages in low and oscillatory shear stress carotid lesions	(24)
Heart failure	In clinical and experimental studies	The higher expression of FMOD was associated with NF-κB stimulation; Altered infiltration of leukocytes in *FMOD-KO* mice	(168)

## Author contributions

Conceptualization, FZ and XP. Literature search and investigation: FZ and YB. Writing—original draft preparation, FZ. Writing—review and editing: XP and X’ R. All authors contributed to the article and approved the submitted version.

## References

[1] Schaefer L. Complexity of danger: the diverse nature of damage-associated molecular patterns. J Biol Chem (2014) 289(51):35237–45. doi: 10.1074/jbc.R114.619304 PMC4271212 25391648

[2] Gong T Liu L Jiang W Zhou R. DAMP-sensing receptors in sterile inflammation and inflammatory diseases. Nat Rev Immunol. 2020 (2):95–112. doi: 10.1038/s41577-019-0215-7 31558839

[3] Chen GY Nuñez G. Sterile inflammation: sensing and reacting to damage. Nat Rev Immunol (2010) 10(12):826–37. doi: 10.1038/nri2873 PMC3114424 21088683

[4] Cao X. Self-regulation and cross-regulation of pattern-recognition receptor signalling in health and disease. Nat Rev Immunol (2016) 16(1):35–50. doi: 10.1038/nri.2015.8 26711677

[5] Fullerton JN Gilroy DW. Resolution of inflammation: a new therapeutic frontier. Nat Rev Drug Discovery (2016) 15(8):551–67. doi: 10.1038/nrd.2016.39 27020098

[6] Marozzi M Parnigoni A Negri A Viola M Vigetti D Passi A. Inflammation, extracellular matrix remodeling, and proteostasis in tumor microenvironment. Int J Mol Sci (2021) 22(15):8102. doi: 10.3390/ijms22158102 34360868 PMC8346982

[7] Nguyen TH Duong CM Nguyen XH Than UTT. Mesenchymal stem cell-derived extracellular vesicles for osteoarthritis treatment: extracellular matrix protection, chondrocyte and osteocyte physiology, pain and inflammation management. CELLS (2021) 10(11):2887. doi: 10.3390/cells10112887 34831109 PMC8616200

[8] Soehnlein O Libby P. Targeting inflammation in atherosclerosis [[/amp]]mdash; from experimental insights to the clinic. Nat Rev Drug Discovery (2021) 20(8):589–610. doi: 10.1038/s41573-021-00198-1 33976384 PMC8112476

[9] Furman D Campisi J Verdin E Carrera-Bastos P Targ S Franceschi C. Chronic inflammation in the etiology of disease across the life span. Nat Med (2019) 25(12):1822–32. doi: 10.1038/s41591-019-0675-0 PMC7147972 31806905

[10] Slavich GM. Understanding inflammation, its regulation, and relevance for health: a top scientific and public priority. Brain Behav Immun (2015) 45:13–4. doi: 10.1016/j.bbi.2014.10.012 PMC4361086 25449576

[11] Ferrucci L Fabbri E. Nbi. inflammageing: chronic inflammation in ageing, cardiovascular disease, and frailty. Nat Rev Cardiol (2018) 15(9):505–22. doi: 10.1038/s41569-018-0064-2 PMC6146930 30065258

[12] Schaefer L Iozzo RV. Biological functions of the small leucine-rich proteoglycans: from genetics to signal transduction. J Biol Chem (2008) 283(31):21305–9. doi: 10.1074/jbc.R800020200 PMC2490788 18463092

[13] Nikitovic D Aggelidakis J Young MF Iozzo RV Karamanos NK Tzanakakis GN. The biology of small leucine-rich proteoglycans in bone pathophysiology. J Biol Chem (2012) 287(41):33926–33. doi: 10.1074/jbc.R112.379602 PMC3464503 22879588

[14] Iozzo RV Schaefer L. Proteoglycan form and function: a comprehensive nomenclature of proteoglycans. Matrix Biol (2015) 42:11–55. doi: 10.1016/j.matbio.2015.02.003 25701227 PMC4859157

[15] Pang X Dong N Zheng Z. Small leucine-rich proteoglycans in skin wound healing. front pharmacol. (2020) 10:1649. doi: 10.3389/fphar.2019.01649 PMC6997777 32063855

[16] Zheng Z Li C Ha P Chang GX Yang P Zhang X. CDKN2B upregulation prevents teratoma formation in multipotent fibromodulin-reprogrammed cells. J Clin Invest. (2019) 129(8):3236–51. doi: 10.1172/JCI125015 PMC6668700 31305260

[17] Schaefer L Babelova A Kiss E Hausser HJ Baliova M Krzyzankova M. The matrix component biglycan is proinflammatory and signals through toll-like receptors 4 and 2 in macrophages. J Clin Invest. (2005) 115(8):2223–33. doi: 10.1172/JCI23755 PMC1174916 16025156

[18] Zeng-Brouwers J Pandey S Trebicka J Wygrecka M Schaefer L. Communications via the small leucine-rich proteoglycans: molecular specificity in inflammation and autoimmune diseases. J Histochem Cytochem (2020) 68(12):887–906. doi: 10.1369/0022155420930303 32623933 PMC7708667

[19] Roedig H Nastase MV Wygrecka M Schaefer L. Breaking down chronic inflammatory diseases: the role of biglycan in promoting a switch between inflammation and autophagy. FEBS J (2019) 286(15):2965–79. doi: 10.1111/febs.14791 30776184

[20] Dong Y Zhong J Dong L. The role of decorin in autoimmune and inflammatory diseases. J Immunol Res (2022) 2022:1283383. doi: 10.1155/2022/1283383 36033387 PMC9402370

[21] Poluzzi C Nastase M-V Zeng-Brouwers J Roedig H Hsieh LT-H Michaelis JB. Biglycan evokes autophagy in macrophages via a novel CD44/Toll-like receptor 4 signaling axis in ischemia/reperfusion injury. Kidney Int (2019) 95(3):540–62. doi: 10.1016/j.kint.2018.10.037 30712922

[22] Vij N Roberts L Joyce S Chakravarti S. Lumican regulates corneal inflammatory responses by modulating fas-fas ligand signaling. Invest Ophthalmol Vis Sci (2005) 46(1):88–95. doi: 10.1167/iovs.04-0833 15623759

[23] Zheng Z Lee KS Zhang X Nguyen C Hsu C Wang JZ. Fibromodulin-deficiency alters temporospatial expression patterns of transforming growth factor-β ligands and receptors during adult mouse skin wound healing. PloS One (2014) 9(3). doi: 10.1371/journal.pone.0090817 PMC3948369 24603701

[24] Shami A Tengryd C Asciutto G Bengtsson E Nilsson J Hultgårdh-Nilsson A. Expression of fibromodulin in carotid atherosclerotic plaques is associated with diabetes and cerebrovascular events. Atherosclerosis (2015) 241(2):701–8. doi: 10.1016/j.atherosclerosis.2015.06.023 26125412

[25] Oldberg A Antonsson P Lindblom K Heinegard D. A collagen-binding 59-kd protein (fibromodulin) is structurally related to the small interstitial proteoglycans PG-S1 and PG-S2 (decorin). EMBO J (1989) 8(9):2601–4. doi: 10.1002/j.1460-2075.1989.tb08399.x PMC401265 2531085

[26] Plaas AHK Neame PJ Nivens CM Reiss L. Identification of the keratan sulfate attachment sites on bovine fibromodulin. J Biol Chem (1990) 265(33):20634–40. doi: 10.1016/S0021-9258(17)30550-1 2243109

[27] Heinegard D Larsson T Sommarin Y Franzén A Paulsson M Hedbom E. Two novel matrix proteins isolated from articular cartilage show wide distributions among connective tissues. J Biol Chem (1986) 261(29):13866–72. doi: 10.1016/S0021-9258(18)67101-7 3759994

[28] Hedbom E Heinegard D. Interaction of a 59-kDa connective tissue matrix protein with collagen I and II. J Biol Chem (1989) 264(12):6898–905. doi: 10.1016/S0021-9258(18)83516-5 2496122

[29] McEwan PA Scott PG Bishop PN Bella J. Structural correlations in the family of small leucine-rich repeat proteins and proteoglycans. J Struct Biol (2006) 155(2):294–305. doi: 10.1016/j.jsb.2006.01.016 16884925

[30] Adini I Ghosh K Adini A Chi ZL Yoshimura T Benny O. Melanocyte-secreted fibromodulin promotes an angiogenic microenvironment. J Clin Invest. (2014) 124(1):425–36. doi: 10.1172/JCI69404 PMC3871226 24355922

[31] Häkkinen L Westermarck J Kähäri VM Larjava H. Human granulation-tissue fibroblasts show enhanced proteoglycan gene expression and altered response to TGF-β1. J Dent Res (1996) 75(10):1767–78. doi: 10.1177/00220345960750101001 8955672

[32] Vélez-Delvalle C Marsch-Moreno M Castro-Muñozledo F Bolivar-Flores YJ Kuri-Harcuch W. Fibromodulin gene is expressed in human epidermal keratinocytes in culture and in human epidermis *in vivo* . Biochem Biophys Res Commun (2008) 371(3):420–4. doi: 10.1016/j.bbrc.2008.04.095 18448071

[33] Gori F Schipani E Demay MB. Fibromodulin is expressed by both chondrocytes and osteoblasts during fetal bone development. J Cell Biochem (2001) 82(1):46–57. doi: 10.1002/jcb.1115 11400162

[34] Sztrolovics R Chen XN Grover J Roughley PJ Korenberg JR. Localization of the human fibromodulin gene (FMOD) to chromosome 1q32 and completion of the cDNA sequence. Genomics (1994) 23:715–7. doi: 10.1006/geno.1994.1567 7851907

[35] Antonsson P Oldberg A. Structure and deduced amino acid sequence of the human fibromodulin gene. Biochim Biophys Acta (1993) 1174:204–6. doi: 10.1016/0167-4781(93)90117-V 8357838

[36] Majava M Bishop PN Hägg P Scott PG Rice A Inglehearn C. Novel mutations in the small leucine-rich repeat protein/proteoglycan (SLRP) genes in high myopia. Hum Mutat (2007) 28(4):336–44. doi: 10.1002/humu.20444 17117407

[37] Scott PG McEwan PA Dodd CM Bergmann EM Bishop PN Bella J. Crystal structure of the dimeric protein core of decorin, the archetypal small leucine-rich repeat proteoglycan. Proc Natl Acad Sci U S A (2004) 101(44):15633–8. doi: 10.1073/pnas.0402976101 PMC524833 15501918

[38] Önnerfjord P Heathfield TF Heinegård D. Identification of tyrosine sulfation in extracellular leucine-rich repeat proteins using mass spectrometry. J Biol Chem (2004) 279(1):26–33. doi: 10.1074/jbc.M308689200 14551184

[39] Paracuellos P Kalamajski S Bonna A Bihan D Farndale RW Hohenester E. Structural and functional analysis of two small leucine-rich repeat proteoglycans, fibromodulin and chondroadherin. Matrix Biol (2017) 63:106–16. doi: 10.1016/j.matbio.2017.02.002 PMC5618690 28215822

[40] Antonsson P Heinegard D Oldberg A. Posttranslational modifications of fibromodulin. J Biol Chem (1991) 266(25):16859–61. doi: 10.1016/S0021-9258(18)55381-3 1885612

[41] Zheng Z Granado HS Li C. Fibromodulin, a multifunctional matricellular modulator. J Dent Res (2023). (2):125–34 doi: 10.1177/00220345221138525 PMC9986681 36515321

[42] Kalamajski S Oldberg Å. Fibromodulin binds collagen type I via glu-353 and lys-355 in leucine-rich repeat 11. J Biol Chem (2007) 282(37):26740–5. doi: 10.1074/jbc.M704026200 17623650

[43] Kalamajski S Oldberg Å. Homologous sequence in lumican and fibromodulin leucine-rich repeat 5-7 competes for collagen binding. J Biol Chem (2009) 284(1):534–9. doi: 10.1074/jbc.M805721200 19008226

[44] Chen S Birk DE. The regulatory roles of small leucine-rich proteoglycans in extracellular matrix assembly. FEBS J (2013) 280(10):2120–37. doi: 10.1111/febs.12136 PMC3651807 23331954

[45] Tillgren V Mörgelin M Önnerfjord P Kalamajski S Aspberg A. The tyrosine sulfate domain of fibromodulin binds collagen and enhances fibril formation. J Biol Chem (2016) 291(45):23744–55. doi: 10.1074/jbc.M116.730325 PMC5095427 27634037

[46] Kalamajski S Liu C Tillgren V Rubin K Oldberg Å Rai J. Increased c-telopeptide cross-linking of tendon type i collagen in fibromodulin-deficient mice. J Biol Chem (2014) 289(27):18873–9. doi: 10.1074/jbc.M114.572941 PMC4081928 24849606

[47] Kalamajski S Bihan D Bonna A Rubin K Farndale RW. Fibromodulin interacts with collagen cross-linking sites and activates lysyl oxidase. J Biol Chem (2016) 291(15):7951–60. doi: 10.1074/jbc.M115.693408 PMC4825002 26893379

[48] Schwarz D Lipoldová M Reinecke H Sohrabi Y. Targeting inflammation with collagen. Clin Transl Med (2022) 12(5):e831. doi: 10.1002/ctm2.831 35604877 PMC9126324

[49] Long G Liu D He X Shen Y Zhao Y Hou X. A dual functional collagen scaffold coordinates angiogenesis and inflammation for diabetic wound healing. Biomater Sci (2020) 8(22):6337–49. doi: 10.1039/D0BM00999G 33025970

[50] Cui S-J Fu Y Liu Y Kou X-X Zhang J-N Gan Y-H. Chronic inflammation deteriorates structure and function of collagen fibril in rat temporomandibular joint disc. Int J Oral Sci (2019) 11(1):2. doi: 10.1038/s41368-018-0036-8 30783108 PMC6381164

[51] Hildebrand A Romaris M Rasmussen LM Heinegard D Twardzik DR Border WA. Interaction of the small interstitial proteoglycans biglycan, decorin and fibromodulin with transforming growth factor β. Biochem J (1994) 302(2):527–34. doi: 10.1042/bj3020527 PMC1137259 8093006

[52] Reis ES Mastellos DC Hajishengallis G Lambris JD. New insights into the immune functions of complement. Nat Rev Immunol (2019) 19(8):503–16. doi: 10.1038/s41577-019-0168-x PMC6667284 31048789

[53] Matzinger P. The danger model: a renewed sense of self. Science (2002) 296(5566):301–5. doi: 10.1126/science.1071059 11951032

[54] Köhl J. The role of complement in danger sensing and transmission. Immunol Res (2006) 34(2):157–76. doi: 10.1385/IR:34:2:157 16760575

[55] Hajishengallis G Reis ES Mastellos DC Ricklin D Lambris JD. Novel mechanisms and functions of complement. Nat Immunol (2017) 18(12):1288–98. doi: 10.1038/ni.3858 PMC5706779 29144501

[56] Ricklin D Hajishengallis G Yang K Lambris JD. Complement: a key system for immune surveillance and homeostasis. Nat Immunol (2010) 11(9):785–97. doi: 10.1038/ni.1923 PMC2924908 20720586

[57] Zipfel PF Skerka C. Complement regulators and inhibitory proteins. Nat Rev Immunol (2009) 9(10):729–40. doi: 10.1038/nri2620 19730437

[58] Kopp A Hebecker M Svobodová E Józsi M. Factor h: a complement regulator in health and disease, and a mediator of cellular interactions. Biomolecules (2012) 2(1):46–75. doi: 10.3390/biom2010046 24970127 PMC4030870

[59] Mahmudpour M Roozbeh J Keshavarz M Farrokhi S Nabipour I. COVID-19 cytokine storm: the anger of inflammation. Cytokine (2020) 133:155151. doi: 10.1016/j.cyto.2020.155151 32544563 PMC7260598

[60] Wang Q Rozelle AL Lepus CM Scanzello CR Song JJ Larsen DM. Identification of a central role for complement in osteoarthritis. Nat Med (2011) 17(12):1674–9. doi: 10.1038/nm.2543 PMC3257059 22057346

[61] Merle NS Church SE Fremeaux-Bacchi V Roumenina LT. Complement system part I - molecular mechanisms of activation and regulation. Front Immunol (2015) 6:1–30. doi: 10.3389/fimmu.2015.00262 26082779 PMC4451739

[62] Pangburn MK Schreiber RD Müller-Eberhard HJ. Formation of the initial C3 convertase of the alternative complement pathway. acquisition of C3b-like activities by spontaneous hydrolysis of the putative thioester in native C3. J Exp Med (1981) 154(3):856–67. doi: 10.1084/jem.154.3.856 PMC2186450 6912277

[63] Ghai R Waters P Roumenina LT Gadjeva M Kojouharova MS Reid KBM. C1q and its growing family. Immunobiology (2007) 212(4–5):253–66. doi: 10.1016/j.imbio.2006.11.001 17544811

[64] Sjöberg A Önnerfjord P Mörgelin M Heinegård D Blom AM. The extracellular matrix and inflammation: fibromodulin activates the classical pathway of complement by directly binding C1q. J Biol Chem (2005) 280(37):32301–8. doi: 10.1074/jbc.M504828200 16046396

[65] Heathfield TF Önnerfjord P Dahlberg L Heinegård D. Cleavage of fibromodulin in cartilage explants involves removal of the n-terminal tyrosine sulfate-rich region by proteolysis at a site that is sensitive to matrix metalloproteinase-13. J Biol Chem (2004) 279(8):6286–95. doi: 10.1074/jbc.M307765200 14660626

[66] Sztrolovics R White RJ Poole AR Mort JS Roughley PJ. Resistance of small leucine-rich repeat proteoglycans to proteolytic degradation during interleukin-1-stimulated cartilage catabolism. Society (1999) 577:571–7. PMC1220192 10215595

[67] Shu CC Flannery CR Little CB Melrose J. Catabolism of fibromodulin in developmental rudiment and pathologic articular cartilage demonstrates novel roles for MMP-13 and ADAMTS-4 in c-terminal processing of SLRPs. Int J Mol Sci (2019) 20(3):1–21. doi: 10.3390/ijms20030579 PMC6386837 30700002

[68] Sjöberg AP Trouw LA Clark SJ Sjölander J Heinegård D Sim RB. The factor h variant associated with age-related macular degeneration (His-384) and the non-disease-associated form bind differentially to c-reactive protein, fibromodulin, DNA, and necrotic cells. J Biol Chem (2007) 282(15):10894–900. doi: 10.1074/jbc.M610256200 17293598

[69] Ward PA. Functions of C5a receptors. J Mol Med (Berl). (2009) 87(4):375–8. doi: 10.1007/s00109-009-0442-7 PMC2754833 19189071

[70] Miyabe Y Miyabe C Murooka TT Kim EY Newton GA Kim ND. Complement C5a receptor is the key initiator of neutrophil adhesion igniting immune complex-induced arthritis. Sci Immunol (2017) 2(7):eaaj2195. doi: 10.1126/sciimmunol.aaj2195 PMC5436313 28529998

[71] He JQ Wiesmann C van Lookeren Campagne M. A role of macrophage complement receptor CRIg in immune clearance and inflammation. Mol Immunol (2008) 45(16):4041–7. doi: 10.1016/j.molimm.2008.07.011 18752851

[72] Csincsi ÁI Kopp A Zöldi M Bánlaki Z Uzonyi B Hebecker M. Factor h-related protein 5 interacts with pentraxin 3 and the extracellular matrix and modulates complement activation. J Immunol (2015) 194(10):4963–73. doi: 10.4049/jimmunol.1403121 PMC4416742 25855355

[73] Csincsi ÁI Szabó Z Bánlaki Z Uzonyi B Cserhalmi M Kárpáti É. FHR-1 binds to c-reactive protein and enhances rather than inhibits complement activation. J Immunol (2017) 199(1):292–303. doi: 10.4049/jimmunol.1600483 28533443

[74] Papp A Papp K Uzonyi B Cserhalmi M Csincsi ÁI Szabó Z. Complement factor h-related proteins FHR1 and FHR5 interact with extracellular matrix ligands, reduce factor h regulatory activity and enhance complement activation. Front Immunol (2022) 13(March):1–15. doi: 10.3389/fimmu.2022.845953 PMC8980529 35392081

[75] Gigli I Fujita T Nussenzweig V. Modulation of the classical pathway C3 convertase by plasma proteins C4 binding protein and C3b inactivator. Proc Natl Acad Sci U. S. A. (1979) 76(12):6596–600. doi: 10.1073/pnas.76.12.6596 PMC411913 293746

[76] Blom AM Kask L Dahlbäck B. CCP1-4 of the C4b-binding protein α-chain are required for factor I mediated cleavage of complement factor C3b. Mol Immunol (2003) 39(10):547–56. doi: 10.1016/S0161-5890(02)00213-4 12431388

[77] Happonen KE Sjöberg AP Mörgelin M Heinegård D Blom AM. Complement inhibitor C4b-binding protein interacts directly with small glycoproteins of the extracellular matrix. J Immunol (2009) 182(3):1518–25. doi: 10.4049/jimmunol.182.3.1518 19155499

[78] Nordström T Blom AM Forsgren A Riesbeck K. The emerging pathogen moraxella catarrhalis interacts with complement inhibitor C4b binding protein through ubiquitous surface proteins A1 and A2. J Immunol (2004) 173(7):4598–606. doi: 10.4049/jimmunol.173.7.4598 15383594

[79] Su Y-C Singh B Riesbeck K. Moraxella catarrhalis: from interactions with the host immune system to vaccine development. Future Microbiol (2012) 7(9):1073–100. doi: 10.2217/fmb.12.80 22953708

[80] Murphy TF Parameswaran GI. Moraxella catarrhalis, a human respiratory tract pathogen. Clin Infect Dis an Off Publ Infect Dis Soc Am (2009) 49(1):124–31. doi: 10.1086/599375 19480579

[81] Laabei M Liu G Ermert D Lambris JD Riesbeck K Blom AM. Short leucine-rich proteoglycans modulate complement activity and increase killing of the respiratory pathogen moraxella catarrhalis. J Immunol (2018) 201(9):2721–30. doi: 10.4049/jimmunol.1800734 PMC6200642 30266767

[82] Soliman AM Barreda DR. Acute inflammation in tissue healing. Int J Mol Sci (2022) 24(1):641. doi: 10.3390/ijms24010641 36614083 PMC9820461

[83] Brinkmann V Reichard U Goosmann C Fauler B Uhlemann Y Weiss DS. Neutrophil extracellular traps kill bacteria. Science (2004) 303(5663):1532–5. doi: 10.1126/science.1092385 15001782

[84] Gillitzer R Goebeler M. Chemokines in cutaneous wound healing. J Leukoc Biol (2001) 69(4):513–21. doi: 10.1189/jlb.69.4.513 11310836

[85] Kolaczkowska E Kubes P. Neutrophil recruitment and function in health and inflammation. Nat Rev Immunol (2013) 13(3):159–75. doi: 10.1038/nri3399 23435331

[86] Segel GB Halterman MW Lichtman MA. The paradox of the neutrophil’s role in tissue injury. J Leukoc Biol (2011) 89(3):359–72. doi: 10.1189/jlb.0910538 PMC6608002 21097697

[87] Yanez DA Lacher RK Vidyarthi A Colegio OR. The role of macrophages in skin homeostasis. Pflugers Arch (2017) 469(3–4):455–63. doi: 10.1007/s00424-017-1953-7 PMC5663320 28233123

[88] Minutti CM Knipper JA Allen JE Zaiss DMW. Tissue-specific contribution of macrophages to wound healing. Semin Cell Dev Biol (2017) 61:3–11. doi: 10.1016/j.semcdb.2016.08.006 27521521

[89] Sica A Mantovani A. Macrophage plasticity and polarization: *in vivo* veritas. J Clin Invest. (2012) 122(3):787–95. doi: 10.1172/JCI59643 PMC3287223 22378047

[90] Chinetti-Gbaguidi G Staels B. Macrophage polarization in metabolic disorders: functions and regulation. Curr Opin Lipidol (2011) 22(5):365–72. doi: 10.1097/MOL.0b013e32834a77b4 PMC3565956 21825981

[91] Wen G Zhang C Chen Q Luong LA Mustafa A Ye S. A novel role of matrix metalloproteinase-8 in macrophage differentiation and polarization. J Biol Chem (2015) 290(31):19158–72. doi: 10.1074/jbc.M114.634022 PMC4521038 26092731

[92] Hans CP Sharma N Sen S Zeng S Dev R Jiang Y. Transcriptomics analysis reveals new insights into the roles of Notch1 signaling on macrophage polarization. Sci Rep (2019) 9(1):1–21. doi: 10.1038/s41598-019-44266-4 31142802 PMC6541629

[93] Shami A Gustafsson R Kalamajski S Krams R Segers D Rauch U. Fibromodulin deficiency reduces low-density lipoprotein accumulation in atherosclerotic plaques in apolipoprotein e-null mice. Arterioscler Thromb Vasc Biol (2013) 33(2):354–61. doi: 10.1161/ATVBAHA.112.300723 23202368

[94] Balan S Saxena M Bhardwaj N. Dendritic cell subsets and locations. Int Rev Cell Mol Biol (2019) 348:1–68. doi: 10.1016/bs.ircmb.2019.07.004 31810551

[95] Dasgupta S Erturk-Hasdemir D Ochoa-Reparaz J Reinecker H-C Kasper DL. Plasmacytoid dendritic cells mediate anti-inflammatory responses to a gut commensal molecule via both innate and adaptive mechanisms. Cell Host Microbe (2014) 15(4):413–23. doi: 10.1016/j.chom.2014.03.006 PMC4020153 24721570

[96] Halasi M Grinstein M Adini A Adini I. Fibromodulin ablation exacerbates the severity of acute colitis. J Inflammation Res (2022) 15:4515–26. doi: 10.2147/JIR.S366290 PMC9374093 35966006

[97] Halasi M Nyska A Rubin L Tal Y Tsokos GC Adini I. Melanocyte-secreted fibromodulin constrains skin inflammation in mice injected with lupus serum. Clin Immunol (2022) 241:109055. doi: 10.1016/j.clim.2022.109055 35640789

[98] Raziyeva K Kim Y Zharkinbekov Z Kassymbek K Jimi S Saparov A. Immunology of acute and chronic wound healing. (2021), 11 (5):700 doi: 10.3390/biom11050700 PMC8150999 34066746

[99] Larouche J Sheoran S Maruyama K Martino MM. Immune regulation of skin wound healing: mechanisms and novel therapeutic targets. Adv Wound Care (2018) 7(7):209–31. doi: 10.1089/wound.2017.0761 PMC6032665 29984112

[100] Landén NX Li D Ståhle M. Transition from inflammation to proliferation: a critical step during wound healing. Cell Mol Life Sci (2016) 73(20):3861–85. doi: 10.1007/s00018-016-2268-0 PMC5021733 27180275

[101] Tottoli EM Dorati R Genta I Chiesa E Pisani S Conti B. Skin wound healing process and new emerging technologies for skin wound care and regeneration. Pharmaceutics (2020) 12(8):735. doi: 10.3390/pharmaceutics12080735 32764269 PMC7463929

[102] Krzyszczyk P Schloss R Palmer A Berthiaume F. The role of macrophages in acute and chronic wound healing and interventions to promote pro-wound healing phenotypes. Front Physiol (2018) 9:419. doi: 10.3389/fphys.2018.00419 29765329 PMC5938667

[103] Chakravarti S. Functions of lumican and fibromodulin: lessons from knockout mice. Glycoconj J (2002) 19(4–5):287–93. doi: 10.1023/A:1025348417078 12975607

[104] Khorasani H Zheng Z Nguyen C Zara J Zhang X Wang J. A quantitative approach to scar analysis. Am J Pathol (2011) 178(2):621–8. doi: 10.1016/j.ajpath.2010.10.019 PMC3070584 21281794

[105] Zheng Z Jian J Velasco O Hsu Cy Zhang K Levin A. Fibromodulin enhances angiogenesis during cutaneous wound healing. Plast Reconstr Surg - Glob Open (2014) 2(12):1–10. doi: 10.1097/GOX.0000000000000243 PMC4292257 25587509

[106] Jian J Zheng Z Zhang K Rackohn TM Hsu C Levin A. Fibromodulin promoted *in vitro* and *in vivo* angiogenesis. Biochem Biophys Res Commun (2013) 436(3):530–5. doi: 10.1016/j.bbrc.2013.06.005 PMC4007216 23770359

[107] Zheng Z Zhang X Dang C Beanes S Chang GX Chen Y. Fibromodulin is essential for fetal-type scarless cutaneous wound healing. Am J Pathol (2016) 186(11):2824–32. doi: 10.1016/j.ajpath.2016.07.023 PMC5222972 27665369

[108] Li CS Yang P Ting K Aghaloo T Lee S Zhang Y. Fibromodulin reprogrammed cells: a novel cell source for bone regeneration. Biomaterials (2016) 83:194–206. doi: 10.1016/j.biomaterials.2016.01.013 26774565 PMC4754141

[109] Zheng Z James AW Li C Jiang W Wang JZ Chang GX. Fibromodulin reduces scar formation in adult cutaneous wounds by eliciting a fetal-like phenotype. Signal Transduct Target Ther (2017) 2:17050 doi: 10.1038/sigtrans.2017.50 29201497 PMC5661627

[110] Zheng Z Nguyen C Zhang X Khorasani H Wang JZ Zara JN. Delayed wound closure in fibromodulin-deficient mice is associated with increased TGF-B3 signaling. J Invest Dermatol (2011) 131(3):769–78. doi: 10.1038/jid.2010.381 PMC4073663 21191417

[111] Lucas T Waisman A Ranjan R Roes J Krieg T Müller W. Differential roles of macrophages in diverse phases of skin repair. J Immunol (2010) 184(7):3964–77. doi: 10.4049/jimmunol.0903356 20176743

[112] Juban G Saclier M Yacoub-Youssef H Kernou A Arnold L Boisson C. AMPK activation regulates LTBP4-dependent TGF-β1 secretion by pro-inflammatory macrophages and controls fibrosis in duchenne muscular dystrophy. Cell Rep (2018) 25(8):2163–76. doi: 10.1016/j.celrep.2018.10.077 30463013

[113] Goldring MB Goldring SR. Articular cartilage and subchondral bone in the pathogenesis of osteoarthritis. Ann N Y Acad Sci (2010) 1192:230–7. doi: 10.1111/j.1749-6632.2009.05240.x 20392241

[114] Han S. Osteoarthritis year in review 2022: biology. Osteoarthr Cartil. (2022) 30(12):1575–82. doi: 10.1016/j.joca.2022.09.003 36150676

[115] Li M Yin H Yan Z Li H Wu J Wang Y. The immune microenvironment in cartilage injury and repair. Acta Biomater. (2022) 140:23–42. doi: 10.1016/j.actbio.2021.12.006 34896634

[116] Ioan-Facsinay A Kloppenburg M. An emerging player in knee osteoarthritis: the infrapatellar fat pad. Arthritis Res Ther (2013) 15(6):225. doi: 10.1186/ar4422 24367915 PMC3979009

[117] Chow YY Chin KY. The role of inflammation in the pathogenesis of osteoarthritis. Mediators Inflamm (2020) 2020:8293921. doi: 10.1155/2020/8293921 32189997 PMC7072120

[118] Wojdasiewicz P Poniatowski ŁA Szukiewicz D. The role of inflammatory and anti-inflammatory cytokines in the pathogenesis of osteoarthritis. Mediators Inflamm (2014) 2014:561459. doi: 10.1155/2014/561459 24876674 PMC4021678

[119] Gill MR Oldberg Å Reinholt FP. Fibromodulin-null murine knee joints display increased incidences of osteoarthritis and alterations in tissue biochemistry. Osteoarthr Cartil. (2002) 10(10):751–7. doi: 10.1053/joca.2002.0527 12359160

[120] Ameye L Aria D Jepsen K Oldberg A Xu T Young MF. Abnormal collagen fibrils in tendons of biglycan/fibromodulin-deficient mice lead to gait impairment, ectopic ossification, and osteoarthritis. FASEB J (2002) 16(7):673–80. doi: 10.1096/fj.01-0848com 11978731

[121] Wadhwa S Embree MC Kilts T Young MF Ameye LG. Accelerated osteoarthritis in the temporomandibular joint of biglycan/ fibromodulin double-deficient mice. Osteoarthr Cartil. (2005) 13(9):817–27. doi: 10.1016/j.joca.2005.04.016 16006154

[122] Embree MC Kilts TM Ono M Inkson CA Syed-Picard F Karsdal MA. Biglycan and fibromodulin have essential roles in regulating chondrogenesis and extracellular matrix turnover in temporomandibular joint osteoarthritis. Am J Pathol (2010) 176(2):812–26. doi: 10.2353/ajpath.2010.090450 PMC2808087 20035055

[123] Jasin HE Taurog JO. Mechanisms of disruption of the articular cartilage surface in inflammation: neutrophil elastase increases availability of collagen type II epitopes for binding with antibody on the surface of articular cartilage. J Clin Invest. (1991) 87(5):1531–6. doi: 10.1172/JCI115164 PMC295233 1708782

[124] Jasin HE Noyori K Takagi T Taurog JD. Characteristics of anti-type ii collagen antibody binding to articular cartilage. Arthritis Rheumatol (1993) 36(5):651–9. doi: 10.1002/art.1780360512 8489543

[125] Mitani Y Honda A Jasin HE. Polymorphonuclear leukocyte adhesion to articular cartilage is inhibited by cartilage surface macromolecules. Rheumatol Int (2001) 20(5):180–5. 10.1007/s002960000098 11518037

[126] Noyori K Plaas A Takagi T Jasin He. Identification of a proteoglycan on the articular cartilage surface that interferes with anti-type II collagen antibody binding. Arthritis Rheumatol (1992) 35(9):S74–4.

[127] Shiomi T Lemaître V D’Armiento J Okada Y. Matrix metalloproteinases, a disintegrin and metalloproteinases, and a disintegrin and metalloproteinases with thrombospondin motifs in non-neoplastic diseases. Pathol Int (2010) 60(7):477–96. doi: 10.1111/j.1440-1827.2010.02547.x PMC3745773 20594269

[128] Hu Q Ecker M. Overview of MMP-13 as a promising target for the treatment of osteoarthritis. Int J Mol Sci (2021) 22(4):1–22. doi: 10.3390/ijms22041742 PMC7916132 33572320

[129] Gobezie R Kho A Krastins B Sarracino DA Thornhill TS Chase M. High abundance synovial fluid proteome: distinct profiles in health and osteoarthritis. Arthritis Res Ther (2007) 9(2):R36. doi: 10.1186/ar2172 17407561 PMC1906814

[130] Kalchishkova N Fürst CM Heinega D Blom AM. NC4 domain of cartilage-specific collagen IX inhibits complement directly due to attenuation of membrane attack formation and indirectly through binding and enhancing activity of complement inhibitors C4B-binding protein and factor h. J Biol Chem (2011) 286(32):27915–26. doi: 10.1074/jbc.M111.242834 PMC3151037 21659506

[131] Xu X Ha P Yen E Li C Zheng Z. Small leucine-rich proteoglycans in tendon wound healing. (2022) (4):202–14. doi: 10.1089/wound.2021.0069 34978952

[132] Fenwick SA Hazleman BL Riley GP. The vasculature and its role in the damaged and healing tendon. Arthritis Res (2002) 4(4):252–60. doi: 10.1186/ar416 PMC128932 12106496

[133] Docheva D Müller SA Majewski M Evans CH. Biologics of tendon repair. Adv Drug Delivery Rev (2015) 84:222–39. doi: 10.1016/j.addr.2014.11.015 PMC4519231 25446135

[134] Millar NL Murrell GAC Mcinnes IB. Inflammatory mechanisms in tendinopathy - towards translation. Nat Rev Rheumatol (2017) 13(2):110–22. doi: 10.1038/nrrheum.2016.213 28119539

[135] Russo V El Khatib M Prencipe G Citeroni MR Faydaver M Mauro A. Tendon immune regeneration: insights on the synergetic role of stem and immune cells during tendon regeneration. Cells (2022) 11(3):434. doi: 10.3390/cells11030434 35159244 PMC8834336

[136] Millar NL Silbernagel KG Thorborg K Kirwan PD Galatz LM Abrams GD. Tendinopathy. Nat Rev Dis Prim. (2021) 7(1):1. doi: 10.1038/s41572-020-00234-1 33414454

[137] Ezura Y Chakravarti S Oldberg A Chervoneva I Birk DE. Differential expression of lumican and fibromodulin regulate collagen fibrillogenesis in developing mouse tendons. J Cell Biol (2000) 151(4):779–87. doi: 10.1083/jcb.151.4.779 PMC2169450 11076963

[138] Svensson L Aszódi A Reinholt FP Fässler R Heinegård D Oldberg Å. Fibromodulin-null mice have abnormal collagen fibrils, tissue organization, and altered lumican deposition in tendon. J Biol Chem (1999) 274(14):9636–47. doi: 10.1074/jbc.274.14.9636 10092650

[139] Jepsen KJ Wu F Peragallo JH Paul J Roberts L Ezura Y. A syndrome of joint laxity and impaired tendon integrity in lumican- and fibromodulin-deficient mice. J Biol Chem (2002) 277(38):35532–40. doi: 10.1074/jbc.M205398200 12089156

[140] Delalande A Gosselin MP Suwalski A Guilmain W Leduc C Berchel M. Enhanced Achilles tendon healing by fibromodulin gene transfer. Nanomedicine Nanotechnology Biol Med (2015) 11(7):1735–44. doi: 10.1016/j.nano.2015.05.004 26048315

[141] Xu X Zhang Y Ha P Chen Y Li C Yen E. A novel injectable fibromodulin-releasing granular hydrogel for tendon healing and functional recovery. Bioeng Transl Med (2023) 8(1):e10355. doi: 10.1002/btm2.10355 36684085 PMC9842059

[142] Bi Y Ehirchiou D Kilts TM Inkson CA Embree MC Sonoyama W. Identification of tendon stem/progenitor cells and the role of the extracellular matrix in their niche. Nat Med (2007) 13(10):1219–27. doi: 10.1038/nm1630 17828274

[143] Rui YF Lui PPY Li G Fu SC Lee YW Chan KM. Isolation and characterization of multipotent rat tendon-derived stem cells. Tissue Eng - Part A. (2010) 16(5):1549–58. doi: 10.1089/ten.tea.2009.0529 20001227

[144] Zhang J Wang JHC. Characterization of differential properties of rabbit tendon stem cells and tenocytes. BMC Musculoskelet Disord (2010) 11:10. doi: 10.1186/1471-2474-11-10 20082706 PMC2822826

[145] Vinhas A Rodrigues MT Gomes ME. Exploring stem cells and inflammation in tendon repair and regeneration. (2018). 1089:37–46 doi: 10.1007/5584_2018_258 30088259

[146] Shen W Chen J Yin Z Chen X Liu H Heng BC. Allogenous tendon stem/progenitor cells in silk scaffold for functional shoulder repair. Cell Transplant. (2012) 21(5):943–58. doi: 10.3727/096368911X627453 22405331

[147] Rui YF Lui PPY Wong YM Tan Q Chan KM. BMP-2 stimulated non-tenogenic differentiation and promoted proteoglycan deposition of tendon-derived stem cells (TDSCs) in vitro. J Orthop Res Off Publ Orthop Res Soc (2013) 31(5):746–53. doi: 10.1002/jor.22290 23238867

[148] Ducy P Karsenty G. The family of bone morphogenetic proteins. Kidney Int (2000) 57(6):2207–14. doi: 10.1046/j.1523-1755.2000.00081.x 10844590

[149] Deng G Li K Chen S Chen P Zheng H Yu B. Interleukin-10 promotes proliferation and migration, and inhibits tendon differentiation via the JAK/Stat3 pathway in tendon-derived stem cells in vitro. Mol Med Rep (2018) 18(6):5044–52. doi: 10.3892/mmr.2018.9547 PMC6236255 30320384

[150] Chen S Deng G Li K Zheng H Wang G Yu B. Interleukin-6 promotes proliferation but inhibits tenogenic differentiation via the janus kinase/signal transducers and activators of transcription 3 (JAK/STAT3) pathway in tendon-derived stem cells. Med Sci Monit (2018) 24:1567–73. doi: 10.12659/MSM.908802 PMC5868364 29547593

[151] Zhang K Asai S Yu B Enomoto-Iwamoto M. IL-1β irreversibly inhibits tenogenic differentiation and alters metabolism in injured tendon-derived progenitor cells in vitro. Biochem Biophys Res Commun (2015) 463(4):667–72. doi: 10.1016/j.bbrc.2015.05.122 PMC4496264 26051275

[152] Tarafder S Chen E Jun Y Kao K Sim KH Back J. Tendon stem / progenitor cells regulate inflammation in tendon healing via JNK and STAT3 signaling. (2017). (9):3991–8 doi: 10.1096/fj.201700071R PMC5572690 28533328

[153] Skålén K Gustafsson M Rydberg EK Hultén LM Wiklund O Innerarity TL. Subendothelial retention of atherogenic lipoproteins in early atherosclerosis. Nature (2002) 417(6890):750–4. doi: 10.1038/nature00804 12066187

[154] Libby P Loscalzo J Ridker PM Farkouh ME Hsue PY Fuster V. Inflammation, immunity, and infection in atherothrombosis: JACC review topic of the week. J Am Coll Cardiol (2018) 72(17):2071–81. doi: 10.1016/j.jacc.2018.08.1043 PMC6196735 30336831

[155] Libby P Buring JE Badimon L Hansson GK Deanfield J Bittencourt MS. Atherosclerosis. Nat Rev Dis Prim (2019) 5(1):56. doi: 10.1038/s41572-019-0106-z 31420554

[156] Shah PK. Molecular mechanisms of plaque instability. Curr Opin Lipidol. (2007) 18(5):492–9. doi: 10.1097/MOL.0b013e3282efa326 17885418

[157] Ström Å Ahlqvist E Franzén A Heinegård D Hultgårdh-Nilsson A. Extracellular matrix components in atherosclerotic arteries of apo E/LDL receptor deficient mice: an immunohistochemical study. Histol Histopathol (2004) 19(2):337–47. doi: 10.14670/HH-19.337. 10.14670/HH-19.337 15024695

[158] Levine B Kalman J Mayer L Fillit Hm Packer M. Elevated circulating levels of tumor necrosis factor in severe chronic heart failure. N Engl J Med (1990) 323(4):236–41. doi: 10.1056/NEJM199007263230405 2195340

[159] Schirone L Forte M Palmerio S Yee D Nocella C Angelini F. A review of the molecular mechanisms underlying the development and progression of cardiac remodeling. Oxid Med Cell Longev (2017) 2017:3920195. doi: 10.1155/2017/3920195 28751931 PMC5511646

[160] Frantz S Falcao-Pires I Balligand JL Bauersachs J Brutsaert D Ciccarelli M. The innate immune system in chronic cardiomyopathy: a European society of cardiology (ESC) scientific statement from the working group on myocardial function of the ESC. Eur J Heart Fail (2018) 20(3):445–59. doi: 10.1002/ejhf.1138 PMC5993315 29333691

[161] Mann DL. The emerging role of innate immunity in the heart and vascular system: for whom the cell tolls. Circ Res (2011) 108(9):1133–45. doi: 10.1161/CIRCRESAHA.110.226936 PMC3084988 21527743

[162] Bajpai G Schneider C Wong N Bredemeyer A Hulsmans M Nahrendorf M. The human heart contains distinct macrophage subsets with divergent origins and functions. Nat Med (2018) 24(8):1234–45. doi: 10.1038/s41591-018-0059-x PMC6082687 29892064

[163] Noutsias M Pauschinger M Schultheiss H K hl U. Phenotypic characterization of infiltrates in dilated cardiomyopathy - diagnostic significance of T-lymphocytes and macrophages in inflammatory cardiomyopathy. Med Sci Monit Int Med J Exp Clin Res (2002) 8(7):CR478–87. 12118194

[164] Adamo L Rocha-Resende C Prabhu SD Mann DL. Reappraising the role of inflammation in heart failure. Nat Rev Cardiol (2020) 17(5):269–85. doi: 10.1038/s41569-019-0315-x 31969688

[165] Mann DL. Innate immunity and the failing heart: the cytokine hypothesis revisited. Circ Res (2015) 116(7):1254–68. doi: 10.1161/CIRCRESAHA.116.302317 PMC4380242 25814686

[166] Waehre A Halvorsen B Yndestad A Husberg C Sjaastad I Nygård S. Lack of chemokine signaling through CXCR5 causes increased mortality, ventricular dilatation and deranged matrix during cardiac pressure overload. PLoS One (2011) 6(4):6–8. doi: 10.1371/journal.pone.0018668 PMC3078912 21533157

[167] Dobner T Wolf I Emrich T Lipp M. Differentiation-specific expression of a novel G protein-coupled receptor from burkitt’s lymphoma. Eur J Immunol (1992) 22(11):2795–9. doi: 10.1002/eji.1830221107 1425907

[168] Andenæs K Lunde IG Mohammadzadeh N Dahl CP Aronsen JM Strand ME. The extracellular matrix proteoglycan fibromodulin is upregulated in clinical and experimental heart failure and affects cardiac remodeling. PLoS One (2018) 13(7):e0201422. doi: 10.1371/journal.pone.0201422. 10.1371/journal.pone.0201422 PMC6063439 30052659

